# T4 Phage Tail Adhesin Gp12 Counteracts LPS-Induced Inflammation *In Vivo*

**DOI:** 10.3389/fmicb.2016.01112

**Published:** 2016-07-14

**Authors:** Paulina Miernikiewicz, Anna Kłopot, Ryszard Soluch, Piotr Szkuta, Weronika Kęska, Katarzyna Hodyra-Stefaniak, Agnieszka Konopka, Marcin Nowak, Dorota Lecion, Zuzanna Kaźmierczak, Joanna Majewska, Marek Harhala, Andrzej Górski, Krystyna Dąbrowska

**Affiliations:** ^1^Bacteriophage Laboratory, Ludwik Hirszfeld Institute of Immunology and Experimental Therapy, Polish Academy of SciencesWroclaw, Poland; ^2^Division of Pathomorphology and Veterinary Forensics, Department of Pathology, Wroclaw University of Environmental and Life SciencesWrocław, Poland

**Keywords:** T4 phage, gp12, short tail fibers, lipopolysaccharide, LPS, inflammation

## Abstract

Bacteriophages that infect Gram-negative bacteria often bind to the bacterial surface by interaction of specific proteins with lipopolysaccharide (LPS). Short tail fiber proteins (tail adhesin, gp12) mediate adsorption of T4-like bacteriophages to *Escherichia coli*, binding surface proteins or LPS. Produced as a recombinant protein, gp12 retains its ability to bind LPS. Since LPS is able to exert a major impact on the immune response in animals and in humans, we have tested LPS-binding phage protein gp12 as a potential modulator of the LPS-induced immune response. We have produced tail adhesin gp12 in a bacterial expression system and confirmed its ability to form trimers and to bind LPS *in vitro* by dynamic light scattering. This product had no negative effect on mammalian cell proliferation *in vitro*. Further, no harmful effects of this protein were observed in mice. Thus, gp12 was used in combination with LPS in a murine model, and it decreased the inflammatory response to LPS *in vivo*, as assessed by serum levels of cytokines IL-1 alpha and IL-6 and by histopathological analysis of spleen, liver, kidney and lungs. Thus, in future studies gp12 may be considered as a potential tool for modulating and specifically for counteracting LPS-related physiological effects *in vivo*.

## Introduction

Bacteriophages that infect Gram-negative bacteria often bind to lipopolysaccharide (LPS) molecules exposed on the surface of the outer membrane. For this action bacteriophages use dedicated attachment proteins. T4-like bacteriophages adsorb to *Escherichia coli* cells by two types of specialized adhesion proteins: long tail fibers and short tail fibers. These two types of adhesins differ in their role during phage infection of bacteria, and thus they differ in the affinity to LPS. Long tail fibers consist of a few proteins that build an elongated flexible structure in which gp37 determines the affinity. Since the role of long tail fiber is “the first screening” and discrimination between susceptible and non-susceptible bacteria, its binding to bacterial receptors is reversible. Short tail fibers are known to bind LPS very firmly since their role is to anchor the phage to a host selected by long tail fibers; they assure a fixed structure during penetration of the bacterial envelope by the tail tube (Miroshnikov et al., 1998; van Raaij et al., 2001a,b; Thomassen et al., 2003).

Short tail fibers of bacteriophage T4 consist of one type of protein, gp12, that forms parallel homo-trimers. These homo-trimers attach to the phage baseplate by the N-terminus while the C-terminus functions as the LPS-binding site (van Raaij et al., 2001a,b; Thomassen et al., 2003). In the phage particle, spatial exposure of gp12 depends on the stage of infection: its LPS-binding region is initially hidden in the baseplate. To expose the LPS-binding region, another structural protein of the phage, gp10, functions as a molecular lever that rotates and extends the hinged short tail fibers, thus facilitating bacterial cell attachment. All six short fibers turn to a perpendicular position that allows for firm binding to LPS molecules (Leiman et al., 2006). Interestingly, isolated gp12, that is expressed as a recombinant protein, retains its ability to bind LPS (van Raaij et al., 2001a,b; Thomassen et al., 2003). This feature has already allowed for practical application of gp12 as an LPS ligand *in vitro*: it serves as a reactant in LPS-removal and LPS-detection kits^1^.

Lipopolysaccharides, also called endotoxins, are able to exert a major impact on the immune response in animals and in humans. LPS is the prototypical pathogen-associated molecular pattern (PAMP); when detected in a living system, LPS triggers a non-specific activation. LPS is recognized by Toll-like receptors (TLRs) and other pattern recognition receptors (PRRs) in both plants and animals (Poltorak et al., 1998; Alexander and Rietschel, 2001; Medzhitov, 2007). TLR4 is present on the surface of monocytes/macrophages, neutrophils, myeloid dendritic cells, mast cells, B lymphocytes, and intestinal epithelium (Sallusto and Lanzavecchia, 2002; Sabroe et al., 2005; Gerondakis et al., 2007). Recognition and immunostimulation by LPS is initialized by the combined extracellular actions of LPS binding protein (LBP), TLR4, MD-2 and CD14 the membrane-bound or soluble forms. The key step is formation of the TLR4-MD-2-LPS complex. This results in the rapid activation of intracellular signaling pathways that are highly similar to the signaling systems of IL-1 and IL-18 (Alexander and Rietschel, 2001). At the systemic level, this reaction means an acute inflammatory response, and it can be a key factor in sepsis and septic shock.

At the same time, effects of bacteriophages on functions of the immune system are gaining increasing interest in the scientific community (Górski et al., 2006, 2012; Łusiak-Szelachowska et al., 2014; Park et al., 2014; Hodyra-Stefaniak et al., 2015). This interest results from safety issues related to practical applications of bacteriophages, but also from the fact that bacteriophages have the intrinsic ability to interact with bacterial products such as LPSs and surface proteins. This ability is mediated by specific phage proteins. Therefore, here we assess LPS-binding phage protein gp12 as a potential modulator of the LPS-induced immune response. This assessment involved initial toxicity testing in cell cultures, and gp12 was further tested for its ability to interfere with LPS-induced immunostimulation *in vivo*.

## Materials and Methods

### Construction of Expression Vector

Genes encoding short tail fiber (*12*) and its chaperone gp57 (*57*) were cloned to the expression plasmid pCDF-Duet1 (Streptomycin resistance; Novagen, USA), which contains two multiple cloning sites (MCS) under a T7lac promoter control. Amplification of gene *12* was conducted using polymerase chain reaction (PCR) and T4 total DNA (Miernikiewicz et al., 2012) as a template. The primers were 12 forward 5′-AAAAGGATCCGATGAGTAATAATACATATCAACACG-3′ and 12 reverse 5′-AAAGCGGCCGCTCATTCTTTTACCTTAATTATG-3′. The *12* gene was inserted into the first multiple cloning site (MCS1) of pCDF-Duet1 via *Bam*HI and *Not*I restriction enzymes (restriction sites are underlined in primer sequences), yielding pCDFg12. Then, the *57* gene was cloned into MCS2 of pCDF-Duet1 by the GeneArt Gene Synthesis system (GeneArt, Thermo Fisher Scientific, Poland), yielding pCDFg12g57. All sequences were verified by automated Sanger sequencing (Genomed, Poland). The obtained vector pCDFg12g57 expresses gp12 with an N-terminal His-Tag and gp57 without any tags.

### Expression and Purification of gp12

The pCDFg12g57 vector was expressed in a bacterial expression system. *E. coli* strain B834(DE3) F- *ompT hsdS_B_* (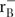 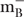) *gal dcm met* (DE3; EMD, Europe) carrying pCDFg12g57 was grown in Luria-Bertani Broth (LB) high salt (10 g/l of NaCl) culture medium (Sigma-Aldrich, Europe or AppliChem, Europe) supplemented with streptomycin at 37°C until OD_600_ reached 0.8. Then, the bacterial culture was cooled down to 18°C, induced by 0.1 mM isopropyl thio-β-D-galactoside (IPTG) and further incubated overnight at 18°C.

Harvested bacteria were suspended in phosphate buffer (50 mM Na_2_HPO_4_, 300 mM NaCl, pH 8), treated with PMSF (1 mM) and incubated on ice for 15 min. The lysis was done by incubation with lysozyme (0.5 mg/ml) for 6–7 h on ice and by the freeze-thaw method (-80°C). The preparation was then supplemented with Mg^2+^ (up to 0.25 mM), DNase (10 μg/ml) and RNase (20 μg/ml), and incubated on ice for 3 h. Fractions were separated by several centrifugations (12 000 rpm, 45 min, 15°C). Soluble fractions were removed while insoluble fractions were suspended in Tris buffer (40 mM Tris–HCl pH 8, 10 mM EDTA) after each centrifugation, and gently mixed for 30 min. The procedure was repeated three times. The last pellets were suspended in phosphate buffer (50 mM Na_2_HPO_4_, 300 mM NaCl, pH 8) and centrifuged in the same conditions as mentioned above. Obtained soluble fractions were filtered through 0.45 μm PVDF filters and further incubated overnight at 10°C with 1 % w/v glycerol. Next, the preparation was supplemented with 25 mM imidazole and incubated with NiNTA agarose (Qiagen, Germany) at room temperature for 2–3 h. The slurry was washed with 5 L of wash buffer I (50 mM NaH_2_PO_4_ × H_2_O, 300 mM NaCl, 25 mM imidazole, 1% glycerol (w/v), 0.05% TWEEN (v/v), pH 8) and then with 5 L of wash buffer II (50 mM NaH_2_PO_4_ x H_2_O, 300 mM NaCl, 25 mM imidazole, 1% glycerol (w/v)). These two steps of washing were conducted with very slow flow, about 200 ml per hour. The last washing step was done with 100 ml of wash buffer III (50 mM NaH_2_PO_4_ x H_2_O, 300 mM NaCl, 100 mM imidazole, 1% glycerol (w/v)). Protein preparations were released from the column using 5–6 sets of 5 ml of elution buffer (50 mM NaH_2_PO_4_ x H_2_O, 300 mM NaCl, 500 mM imidazole, 4% glycerol (w/v)). The whole process was monitored by SDS-PAGE. Elutions were combined according to their purity and concentrated 2–5 times on Vivaspin centrifuge concentrators (Sartorius, Poland) at 18–20°C. Next, LPS was removed using EndoTrap Blue (Miernikiewicz et al., 2012). The final preparation was dialyzed against PBS at room temperature and filtered through 0.22 μm PVDF filters. Its concentration was determined by Lowry assay. Endotoxin level of the purified gp12 was assessed using the EndoLisa Endotoxin Detection kit (Dąbrowska et al., 2014).

### Particle Size Distribution

Lipopolysaccharide was isolated from *E. coli* liquid cultures as described (Miernikiewicz et al., 2013). It was diluted with deionized water and dispersed in an ultrasonic bath. Dispersion (homogeneity) was controlled by particle size distribution measurement; particle size distribution was analyzed by the dynamic light scattering (DLS) method using Malvern Zetasizer NanoZS (Malvern Instruments, UK). The same system was used for detection of LPS-gp12 interactions: particle size distribution was measured in solutions gp12 (10 μg/ml), LPS (10 μg/ml) or a mixture of gp12 (10 μg/ml) and LPS (10 μg/ml). All samples were diluted with deionized water and offset for 1 min prior to each measurement. Albumin was used as a control. All experiments were done at 25°C. The measurements were repeated five times.

### Cytotoxicity

The influence of gp12 on growth and viability of murine and human cells was investigated. Tests were done on murine fibroblasts (Balb3T3) and on human skin microvascular endothelial cells (HSkMEC). Cells (10^4^ cells/well) were grown in 96-well plates for 24 h at 37°C, 5% CO_2_. Then, cells were supplemented with various concentrations of gp12 (1, 10, 100 μg/ml) and incubated for 72 h. PBS treated cells served as a normal cell growth control (solvent control). Next, the sulforhodamine B (SRB) test was done. Briefly, 50 μl of 50% trichloroacetic acid was added to each well and incubated for 1 h at 4°C. Then, plates were washed five times with H_2_O, treated with 50 μl of 0.4% sulforhodamine and incubated for 30 min at room temperature. 1% acetic acid was used to wash plates and 10 nM Tris to dissolve precipitated sulforhodamine. Measurement of cell viability was done at 540 nm using a microplate reader (Gen5, Data Analysis Software).

### Animal Model

All animal experiments were performed according to EU Directive 2010/63/EU for animal experimentations and were approved by the 1st Local Committee for Experiments with the Use of Laboratory Animals, Wrocław, Poland. The female C57Bl6/J (6–10 weeks) mice were purchased from the Center of Experimental Medicine, Medical University of Białystok, Poland, and bred under specific pathogen free (SPF) conditions in the Animal Breeding Center of the Institute of Immunology and Experimental Therapy (IIET).

Female C57Bl6/J mice (*N* = 6) were injected intraperitoneally with 100 μg/mouse of gp12, 1 mg/kg of LPS or 100 μg/mouse of gp12 simultaneously with 1 mg/kg of LPS. Control mice were inoculated with PBS. After 3 and 7 h murine blood was collected from the tail vein, whilst after 24 h murine blood was collected from the orbital plexus vein into heparinized tubes. All bleeding procedures were done under anesthesia. Serum was separated from the blood by double centrifugation at 2250 × *g* and used for the ELISA assay. Then animals were sacrificed by cervical dislocation and the following organs were excised, for histopathological examination: lungs, liver, spleen, and kidneys. All experiments were repeated three times. A representative experiment is presented.

### Cytokine Assay

The progress of the inflammatory reaction in the murine blood was monitored. Concentrations of IL-1α and IL-6 were measured by commercially available ELISA kits (PeproTech). Mean values per group of animals are presented.

### Histopathology

For histopathological examination, the murine organs (lungs, liver, spleen, and kidneys) were fixed in 7% formaldehyde for 48 h. Later, samples were embedded in paraffin blocks. Histological slides of 4 μm thickness were prepared and counterstained with hematoxylin and eosin (HE), and examined microscopically. All sections were analyzed by researchers blind to the experimental groups of the samples.

### Statistics

Statistical analysis was done by ANOVA, the Kruskal–Wallis test and ANOVA with Bonferroni and Holm multiple comparison calculation, with significance level *p* = 0.05. The Statistica 8.0 software package was applied (StatSoft, Inc. STATISTICA data analysis software system, version 8.0)^2^.

## Results

### Expression and Purification of gp12

**FIGURE 1:**
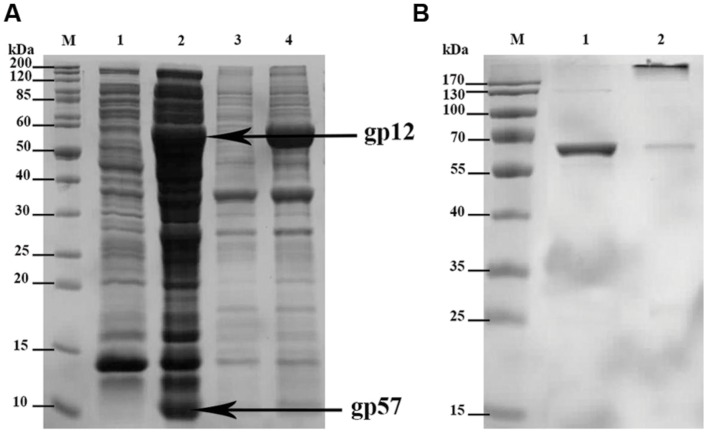
**SDS-PAGE of: (A)** expressed recombinant His-tagged gp12 and gp57; overexpressed proteins are marked with arrows; M-molecular weight marker; 1-soluble fraction of the culture before induction (control); 2-soluble fraction of the culture after induction (expression); 3-insoluble fraction of the culture before induction (control); 4-insoluble fraction of the culture after induction (expression). **(B)** gp12 final preparation as trimeric and monomeric; M – molecular weight marker; 1 – heated gp12 (denatured protein) as monomeric (57.5 kDa); 2 – non-heated gp12 as trimeric (172.5 kDa).

The short tail fibers of bacteriophage T4 are well known for their LPS binding capability. This ability depends, however, on correct gp12 quaternary structure meaning that phage adhesin has to form its trimeric structure to be able to bind LPS. Proper folding of gp12 was reported only in the presence of another T4 phage protein, gp57. Its function as a molecular chaperone cannot be substituted by host protein overexpression, e.g., GroEL/ES. Gp57 increases folding efficacy and production efficiency of gp12, and it concurrently inhibits insoluble aggregate formation by this phage adhesin (Burda and Miller, 1999). Thus, we cloned genes *12* and *57* of T4 phage to pCDF-Duet1, the expression vector that enables simultaneous production of two proteins in bacterial expression systems (**Figure 1A**).

Co-expression of gp12 with its natural chaperonin gp57 resulted in proper trimeric structure of the obtained product as verified by SDS-PAGE. Verification was done according to King and Laemmli (1971), who demonstrated that properly folded, trimeric gp12 resisted dissociation by SDS at ambient temperature, whereas heating caused monomerization of the polypeptide chains (King and Laemmli, 1971). The molecular weight of trimeric gp12 is 172.5 kDa, while that of monomeric gp12 is 57.5 (**Figure 1B**).

### Binding of gp12 to Lipopolysaccharide *In Vitro*

**FIGURE 2:**
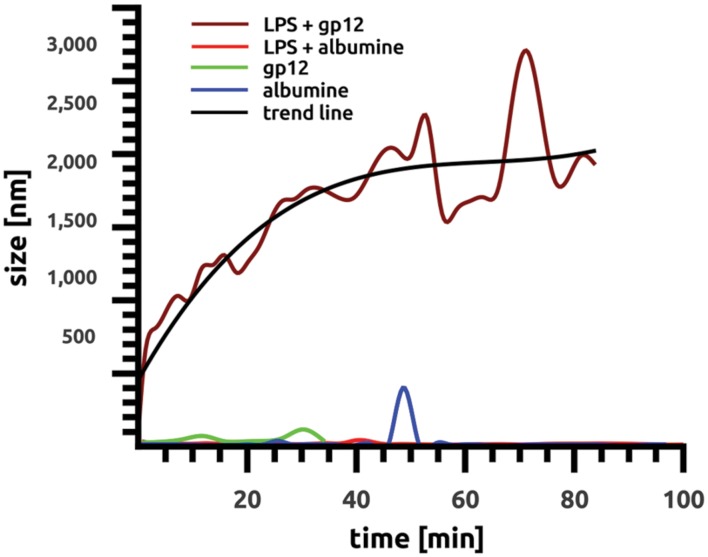
**Binding of gp12 to lipopolysaccharide *in vitro* over time.** Binding was identified as changes in average particle size in solution by the dynamic light scattering (DLS) method using Malvern Zetasizer NanoZS (Malvern Instruments, UK). All samples were diluted with deionized water, and size distribution was measured at 25°C.

The ability of purified recombinant gp12 to bind LPS was tested *in vitro* by particle size distribution measurement in LPS solution. This method employed DLS, and it allowed for detection of a relative increase in average size of aggregating molecules in solutions. Purified gp12 solution as well as LPS solution was dominated by small molecules, 51 nm and 95.45 nm, respectively; these values were stable in time. However, after mixing gp12 with LPS the average size of molecules in the solution markedly increased to 1980 nm (**Figure 2**). These results suggest that recombinant gp12 was able to bind and form complexes with LPS. Formation of these complexes was clearly visible within a few minutes, and it seemed to be stabilized after approximately 45 min, with the average diameter of complexes being approx. 2000 nm (**Figure 2**).

### Safety Testing of gp12 on Cell *In Vitro* Cultures

**FIGURE 3:**
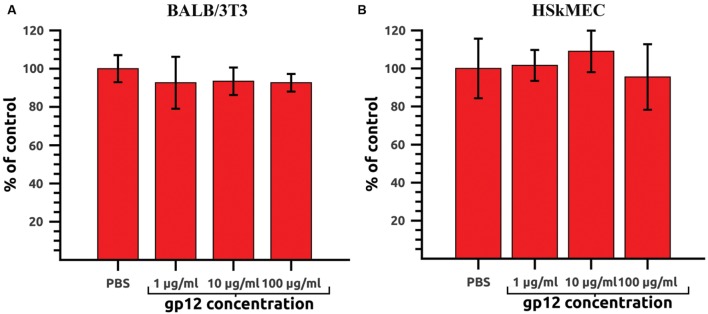
**Proliferation of gp12-treated mammalian cells *in vitro*.** **(A)** Murine fibroblasts (BALB/3T3) treated with 1, 10, or 100 μg/ml of gp12 for 72 h. PBS served as a control. **(B)** Human skin microvascular endothelial cells (HSkMEC) treated with 1, 10, or 100 μg/ml of gp12 for 72 h. PBS served as a control.

Recombinant gp12, intended for *in vivo* application, was first tested on mammalian cell lines *in vitro* to exclude its direct toxic effects on cells. Potential harmful effects were assessed in a proliferation assay on murine fibroblasts (BALB/3T3) and HSkMEC. Both cell lines were treated with gp12 in cultures (doses: 100, 10, or 1 μg of the tested protein per ml). After 72 h of incubation, cell viability was assessed by the SRB method. No toxic or antiproliferative effects of gp12 were detected in the cells (**Figures 3A,B**).

### Effect of gp12 on LPS-Induced Inflammation *In Vivo*

The effect of gp12 on LPS-induced inflammation *in vivo* was investigated in mice by comparison of serum inflammatory markers (cytokines). Cytokines that are typical markers of inflammation induced by LPS are IL-1α and IL-6. Here we compared IL-1α and IL-6 in mice treated with LPS (1 mg/kg) or with purified gp12 (100 μg/mouse) or treated both with LPS (1 mg/kg) and purified gp12 (100 μg/mouse). Control mice were treated with PBS.

**FIGURE 4:**
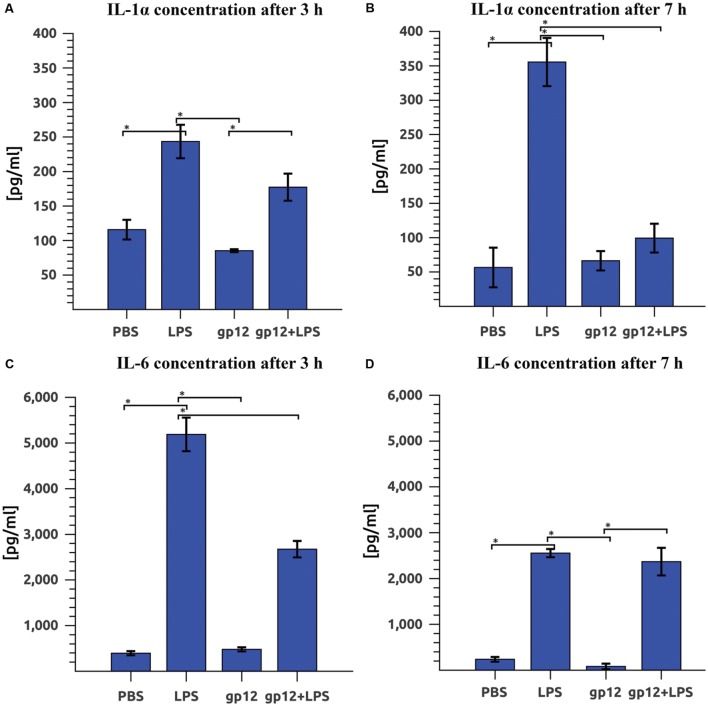
**Effect of phage protein gp12 on: **(A)** IL-1α concentration in mice 3 h after treatment.** **(B)** IL-1α concentration in mice 7 h after treatment; **(C)** IL-6 concentration in mice 3 h after treatment; **(D)** IL-6 concentration in mice 7 h after treatment. ^∗^*p* = 0.05.

Immediate application of gp12 to mice challenged with LPS resulted in a small reduction of serum IL-1α 3 h after the treatment (27% reduction, insignificant) and in a substantial reduction 7 h after the treatment: 72% (*p* = 0.002). In this case, the serum level of IL-1α in mice challenged with LPS and treated with gp12 was similar to that observed in control mice (**Figures 4A,B**). The second marker of inflammation, IL-6, was also reduced in LPS-challenged mice treated with gp12 (in comparison to non-treated animals). This reduction was 48% (*p* = 0.001) 3 h after the treatment; however, no important effect was observed 7 h after the treatment (**Figures 4C,D**). 24 h after the challenge with LPS no significant differences were noted, while serum levels of the tested cytokines were normalized after that time (data not shown). Importantly, in mice treated with gp12 alone no pro-inflammatory activity of this protein was observed and no visible adverse effects of the treatment were noted in these animals (**Figure 4**).

**FIGURE 5:**
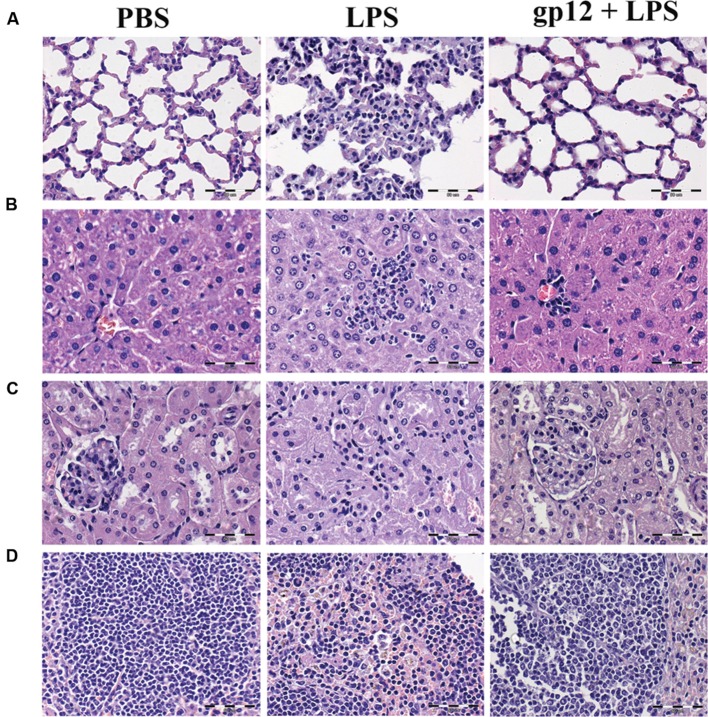
**Histopathological examination of selected animal tissues.** **(A)** lungs; **(B)** liver; **(C)** kidneys; **(D)** spleen.

Further, leukocyte infiltration into selected tissues was examined in these animals by histological microscopy (24 h after the induction). As presented in **Figure 5**, infiltration of leukocytes to lungs, liver and spleen was markedly increased in mice treated with LPS when compared to control (PBS treated) mice, which indicated an inflammatory reaction of the immune system. However, in mice treated with recombinant gp12 immediately after LPS injection, leukocyte infiltration in the investigated tissues was minor (**Figure 5**). Gp12 significantly decreased inflammatory infiltrate induced by LPS in liver and spleen (**Figure 5**). No harmful effects of gp12 was observed in tissues of mice treated with gp12 alone. These observations suggest that gp12 may counteract the pro-inflammatory effect of LPS *in vivo*.

## Discussion

Bacteriophage T4 tail adhesin gp12 was produced in native conformation as a recombinant protein in a bacterial expression system with the chaperone gp57. Similarly to its natural prototype, this protein was able to form trimers and to bind LPS *in vitro*, as shown by DLS (**Figure 2**). Such a product was not harmful for cell proliferation in cell cultures *in vitro*, nor were there any evident harmful effects on living mice (100 μg/mouse). It was further used in combination with LPS in a murine model to assess whether binding of gp12 to LPS might decrease the ability of LPS to induce inflammation *in vivo*. As revealed by serum cytokine assay and by examination of leukocyte infiltration into spleen, liver, kidney, and lungs, gp12 was able to counteract the inflammatory response to LPS *in vivo*.

LPS consists of a poly- or oligosaccharide region that is anchored in the outer bacterial membrane by a specific carbohydrate lipid moiety termed lipid A. The lipid A component is commonly considered as the primary immunostimulatory center of LPS (Alexander and Rietschel, 2001) while phage short tail fibers bind to the core region of LPS (van Raaij et al., 2001a,b). However, immunoactivation by LPS in mammalian systems depend on the LPS form. One can find “classical” strongly agonistic (highly endotoxic) forms of LPS, but several have been identified that display comparatively low or even no immunostimulation for a given mammalian species. Some members of the latter more heterogeneous group are capable of antagonizing the effects of strongly stimulatory LPS/lipid A forms (Alexander and Rietschel, 2001). These observations suggest that details of LPS structure determine its ability to induce an inflammatory response. In light of this, binding of gp12 to the LPS molecule may affect the LPS structure sufficiently to decrease its ability to induce immunostimulation.

Moreover, the TLR4-MD-2-LPS complex has been crystallized and its structure has been determined at 3.1 Å resolution by Park et al. (2009), thus revealing involvement of the whole LPS molecule in the reaction with the mammalian receptor complex. Binding of LPS induces the formation of a symmetrical dimer of two TLR4-MD-2-LPS complexes. The LPS bound to MD-2 directly bridges the two TLR4 molecules. Five of the six lipid chains in LPS are completely buried inside the MD-2 pocket, but the sixth is partially exposed to the surface of MD-2 and forms a hydrophobic interaction with TLR4. Hydrophilic side chains in the surrounding regions of MD-2 and TLR4 support the hydrophobic core of the interface by forming hydrogen bonds and ionic interactions (Gay and Gangloff, 2007; Kim et al., 2007; Park et al., 2009). The molecular weight of gp12 (56 kDa) is considerable in comparison to TLR4 (approx. 95 kDa). We hypothesize that binding of phage protein gp12 to the hydrophilic core disturbs its function in formation of TLR4-MD-2-LPS complexes, and may preclude a signaling pathway that could lead to immunostimulation.

We propose interaction of the phage tail adhesin gp12 with LPS as a potential modulator of LPS-induced inflammatory effects. It is necessary to emphasize that sepsis or septic shock may have different etiology. However, in many cases LPS is the key factor triggering harmful physiological processes. Elucidation of structure-activity correlations in LPS has contributed to understanding of both immunostimulatory and toxic septic processes, and it allowed for development of new pharmacological and immunostimulatory strategies in infectious and malignant diseases. Thus, in future studies gp12 may be considered as a potential tool for modulation and specifically for counteracting LPS-related physiological effects *in vivo*.

## Author Contributions

PM planned the experiments, executed data processing, and analysis, participated in writing the manuscript, executed most of the laboratory work: gene cloning, protein expression and purification, LPS preparation, dynamic light scattering, protein toxicity testing *in vitro*, development and testing in animal model, cytokine tests. WK and AnK participated in protein expression and purification, cytokine testing and work with animals. RS participated in protein expression and purification, LPS preparation and dynamic light scattering. PS participated in protein expression and purification and in protein toxicity testing *in vitro*. MN executed and analyzed histopathological assay. KH-S, AgK, DL, ZK, and JM participated in laboratory work, in work with animals and in data processing. MH prepared graphics presented in the manuscript. AG consulted immunological aspects of the work and reviewed the manuscript. KD guided and supervised the work and analysis of results, wrote the manuscript, participated in laboratory work and in work with animals.

## Conflict of Interest Statement

Authors have filed a patent application for gp12 application *in vivo* (in progress).

## Footnotes

1 http://www.hyglos.de/en/products-services/products/endotoxin-removal.html (accessed December 6, 2015).

2 StatSoft, Inc: STATISTICA (data analysis software system), version 8.0. Available at: http://www.statsoft.com (accessed April 20, 2015).
